# Effectiveness of the European Natura 2000 network to sustain a specialist wintering waterbird population in the face of climate change

**DOI:** 10.1038/s41598-020-77153-4

**Published:** 2020-11-20

**Authors:** Dominik Marchowski, Łukasz Ławicki, Anthony D. Fox, Rasmus D. Nielsen, Ib K. Petersen, Menno Hornman, Leif Nilsson, Fredrik Haas, Johannes Wahl, Jan Kieckbusch, Hans W. Nehls, Neil Calbrade, Richard Hearn, Włodzimierz Meissner, Niamh Fitzgerald, Leho Luigujoe, Marco Zenatello, Clemence Gaudard, Sven Koschinski

**Affiliations:** 1 Ornithological Station, Museum and Institute of Zoology, Polish Academy of Sciences, ul. Nadwiślańska 108, 80-680 Gdańsk, Poland; 2 West Pomeranian Nature Society, Szczecin, Poland; 3 Department of Bioscience, Aarhus University, Kalø, Grenåvej 14, 8410 Rønde, Denmark; 4 Sovon, Dutch Centre for Field Ornithology, Nijmegen, The Netherlands; 5 Department of Biology, Lund University, Lund, Sweden; 6 Federation of German Avifaunists (DDA), An den Speichern 2, 48157 Münster, Germany; 7 Ornithological Working Group Schleswig-Holstein, Lange Reihe 14 d, 24244 Felm, Germany; 8 Ornithological Working Group Mecklenburg-Vorpommern, Bertold-Brecht-Straße 19, 18106 Rostock, Germany; 9 Wetland Bird Survey, British Trust for Ornithology, The Nunnery, Thetford, Norfolk IP24 2PU UK; 10 Wildfowl & Wetlands Trust (WWT), Slimbridge, Glos. GL2 7BT UK; 11 Avian Ecophysiology Unit, Department of Vertebrate Ecology and Zoology, Faculty of Biology, The University of Gdańsk, Gdańsk, Poland; 12 I-WeBS Office, BirdWatch Ireland, Unit 20, Block, D, Bullford Business Campus, Kilcoole, Co. Wicklow Republic of Ireland; 13 Institute of Agricultural and Environmental Sciences, Estonian University of Life Sciences, Tartu, Estonia; 14 Istituto Superiore per la Protezione e la Ricerca Ambientale, Ozzano Emilia, Italy; 15 Ligue pour la Protection des Oiseaux (LPO) BirdLife, Rochefort, France; 16 Meereszoologie, Nehmten, Germany

**Keywords:** Ecology, Zoology, Climate sciences

## Abstract

Analysis of coordinated Greater Scaup (*Aythya marila)* count data from the last 30 years showed a 38.1% decrease in wintering numbers in North-West Europe, from 309,000 during 1988–1991 to *c.*192,300 individuals during 2015–2018. Annual trends in wintering numbers differed throughout the range. Numbers decreased in the UK, Ireland, and in the Netherlands, while numbers were stable in Denmark. Germany, Poland, Sweden, and Estonia showed increasing numbers, suggesting a shift in the distribution of the species within its wintering grounds towards the east and north. Higher temperatures in northern and eastern areas were correlated with the range shift of the wintering distribution. Deaths from bycatch drowning of Scaup in fishing gear have significantly decreased in recent decades in the Netherlands, where currently the greatest threat is considered the deterioration of food resources. The increasing concentration of wintering Scaup in coastal Poland and Germany (where lack of effective implementation of conservation measures fail to protect the species from the impacts of bycatch and declining food quality) pose major threats to the entire population.

## Introduction

For most avian species, the non-breeding “survival” period takes up the majority of the annual cycle^1,2^. Migratory species spend extended periods on wintering or staging sites, and some of them do so in large flocks. The result of such a strategy can be that a significant percentage of an entire population may be restricted to highly limited geographical areas^3^. One such area for Arctic/sub-Arctic breeding sea ducks is the Baltic Sea, which constitutes one of the most important wintering areas for sea ducks in the entire Palearctic^4^. Bird populations with large non-breeding concentrations within limited geographical areas, which also breed at low concentrations across vast areas, present major challenges to effective conservation for countries where they winter. Although in an evolutionary context, such strategies will have favoured enhanced fitness, in the modern world, human activities threaten these species, for instance, through marine pollution, fishery bycatch, hunting, or disturbance from water-sports and offshore wind farms, all of which can adversely affect waterbirds occurring in dense aggregations. On the other hand, the advantage of such a life strategy is that appropriate implementation of targeted spatially-explicit conservation measures can bring rapid positive results^5–7^.

Waterbirds are considered as some of the best indicators of the state of the aquatic environment^8^, with observed densities proportional to habitat quality, profitability and inversely proportional to disturbance or predation risk. However, high concentrations may also result from a severe lack of suitable habitats elsewhere, pressing waterbirds to aggregate in inferior habitats where they may be exposed to increased mortality^9^. One of the objectives of the network of Natura 2000—Special Protection Areas (SPA) designated under the Birds Directive (Council Directive 2009/147/EC) in European Union (EU)—is to protect a cohesive network of important areas for populations of a given migratory or conservation priority (Annex I of the Birds Directive) species. Where SPA designation specifically protects important habitats for a given species, this is mentioned in the formal citation of the site and is referred to as being a *qualifying species* in this SPA.

In this article, we consider the Greater Scaup (*Aythya marila*, hereafter Scaup), a species that reflects the challenges facing the protection of many other migratory waterbirds within the Natura 2000 network under the threat of climate change. Scaup breed in the forest-tundra and tundra region, from Iceland through to upland northern Scandinavia and across the Russian tundra^10^. The small Icelandic breeding population winters mostly in Ireland and the UK^11^ but Russian and Fennoscandian birds winter from the Baltic, through the North Sea coasts, also reaching the UK and Ireland in winter^12^. Scaup generally is not found on inland freshwater bodies often used by the related Tufted Duck (*Aythya fuligula*) or Pochard (*Aythya ferina*). Although not strictly a diving duck of the open sea in the same sense as species such as the Velvet Scoter (*Melanitta fusca*) or Long-tailed Duck (*Clangula hyemalis*), a large proportion of wintering Scaup occurs in coastal marine European waters^13^. Scaup typically exploit transitional waters, mainly coastal lagoons, shallow sea bays, and lakes subject to brackish influence^2^. The relative rarity of such habitat results in significant concentrations of the species (sometimes exceeding 100,000 individuals^14^), especially in shallow waters where this benthic feeding species can dive to feed on abundant bivalve food items, such as Blue Mussel *Mytilus edulis* and Zebra Mussel *Dreissena polymorpha*^2^.

Shifts in the wintering distributions of other diving duck species in response to climate change have been documented^15^ and the importance of protected areas under this scenario has been emphasised^16^. In this article we: (i) assess changes in the past and current status and distribution of North-West Europe wintering Scaup over the last 30 years; (ii) test whether its wintering range has shifted in response to climate change; (iii) review current pressures facing this species on the wintering grounds and assess potential new threats or benefits caused by range shifts and occupancy of new areas; and finally (iv) assess the degree of protection and effectiveness of conservation measures for Scaup concentrations provided by the European Natura 2000 network.

## Methods

### Data collection

We analysed long-term mid-winter count data for Scaup from the most important wintering resorts in Denmark, Sweden, Estonia, Poland, Germany, the Netherlands, France, the United Kingdom, Ireland and Italy, countries that regularly support the greatest numbers of the species in the non-breeding period. These data derive from long-term national waterbird monitoring programmes carried out on the same discrete wetlands or sections of marine coastline, which allows changes in overall population size to be tracked^17^. Additional counts from other European countries where survey coverage is more modest or sporadic were not considered here.

Exceptionally long-term data of wintering Scaup were available from the United Kingdom and Sweden back to 1967 (which we analyse here as separate case studies), but to ensure consistency of coverage at major sites, we used data from 1988 to 2018 inclusive to generate the overall trend in numbers for the entire wintering population in North-West Europe. This period slightly exceeds the three-generation periods for Scaup^18^, so the trend over such a time period fulfils the IUCN criteria for determining a species’ red list status. We organised a targeted coordinated count of wintering Scaup in Europe in January 2015 to count all sites where Scaup were known to occur. For regular monitoring and for the 2015 survey, we used the standard methodology for counting waterbirds during the non-breeding period^17^, supplementing shore-based counts with surveys from ship and aircraft where appropriate. All the count data are from mid-winter January counts, the date of which is determined by the coordinating body (Wetlands International).

### Estimation and categorization of trends

The average population growth rate (λ) obtained by fitting a generalized estimating equation model (GEE) to count data with Poisson error terms to categorise trends, where site counts in a given year are assumed to be the result of a site and year effect, estimating the average λ from the exponential model and its 95% confidence intervals^19^. The model takes account of serial autocorrelation between counts at the same site in different years and enables the estimation of missing values (i.e. at uncounted sites), indicating the uncertainty of estimation by 95% confidence intervals^19^.

Estimation of numbers and λ were performed in TRIM software (Trends and Indices for Monitoring data, ver. 3.54), developed by Statistics Netherlands^19^. Counts are point estimators and show the ratio of the number of a given species in a given year relative to the abundance level reached in the first year of monitoring. The measure of uncertainty of estimating the population size for each year is characterised by a standard error (translating into confidence intervals: CI ≈ 1.96 × SE) and depends on the “natural” variability of results and the amount of data. For sparsely distributed and/or low numbers, the assessment of abnormalities will be affected by a large error, which makes it virtually impossible to detect (small) abnormalities. Since the criteria for trend classification used in TRIM are directly related to the width of the confidence interval, the greater the error of estimation, the smaller the chance that the trend will be classified as significant. This may be despite the fact that in reality changes in numbers have taken place, in other words, directional changes in population size may remain undetected when the precision of indicator estimates is low^19^. The multiplicative overall slope estimate in TRIM is converted into six categories (Table S1).

### The effect of temperature on the number of birds

Observations at a major Baltic wintering site for Scaup (e.g. Odra estuary) indicates that this species is sensitive to ice-cover. The larger the degree of ice-cover, the fewer birds^20^. The ice-cover effect is not applicable in countries where this phenomenon does not occur or is very rare in winter. For this reason, we analysed temperature effects on Scaup abundance where ice-cover episodes persistently occur, namely in three countries in the east and north of wintering range: Poland, Sweden and Estonia. For these countries, we modelled the number of birds using the sum of days with daily temperatures below zero in the first half of January. This measure should represent the degree of ice-cover, the greater the number of days with temperatures below zero, the greater the likelihood of the waters being frozen. Temperature data came from the website: https://en.tutiempo.net/, from measuring stations in Stockholm/Arlanda (Sweden), Świnoujście (Poland) and Tartu/Ulenurme (Estonia). We fitted a generalized linear regression model to the data using the IBM SPSS Statistics 25 package, including numbers of birds (‘birds no.’) as the dependent variable, with the number of days with temperature below zero in the first half of January (‘ice’) and the year in which the count was carried out (‘year’) as explanatory variables.

### Overview of Natura 2000—Special Protection Areas

To assess the effectiveness of SPAs for conserving Scaup along their flyway, we overlaid the distribution of this species based on collected non-breeding season count data for the purpose of this study, upon the geographical extent of existing SPAs using the website: https://natura2000.eea.europa.eu/. We determined the extent to which SPAs included areas regularly used by Scaup and the degree to which unprotected sites supported the species. In areas where regular Scaup concentrations overlapped with SPAs, we analysed the documentation (Standard Data Form—SDF) relating to Scaup which is held at the site. We checked whether Scaup was specifically listed as a *qualifying species* and whether a management plan (MP) existed for this SPA. This analysis focused on all areas that are important for the survival of the whole population, omitting only less important areas where birds appeared irregularly, as single individuals or small groups, not exceeding 30 individuals during the years 2015–2018. We used chi-square statistics to test for statistical significance.

Given the unique tendency for Scaup to concentrate in large numbers within relatively few small areas, we conducted spatial analysis based on their density and distribution aimed at identifying the numerically most important areas and the degree of isolation of Scaup wintering areas. By setting the map scale so that the entire European range of the Scaup wintering grounds (1:12,500,000) was visible, we used the point cluster and point displacement functions within the QGIS ver. 3.4.8. software to establish the degree of clustering between Scaup resorts, based on the distribution of these points, regardless of how many birds were counted at each point. We then carried out a more advanced spatial analysis, which also took into account the numerical importance of each site, weighting the points by count size using kernel density estimation (KDE). The set of points generated from the coordinated count in 2015 was processed in the CRIMESTAT 4.02 software^21^. Following Marchowski and Leitner^9^, we used the weighted KDE analysis: (1) a smoothing parameter (bandwidth length) based on the average distance between all points in the sample and (2) a quartic kernel function with a fixed interval bandwidth length. Based on this analysis, we were able to identify the most important areas for Scaup, which we assigned as “Core wintering areas”. The analysis was then repeated using KDE but without any weighting of the points to indicate all areas of concentration, even those of lesser numerical importance.

Following BirdLife International^22^ criterion A4i, developed after Ramsar (Ramsar Convention on Wetlands) criterion B6, sites considered important for a given population of a species must regularly hold 1% or more of the biogeographic population to qualify as being of international importance. This threshold has also been adapted by EU as a criterion for the establishment of SPAs^23^. The results of this work will therefore determine the current estimated size of the North-West Europe Scaup population and a new 1% threshold for the identification of important areas.

## Results

### Estimated population size and trends

**Figure 1:**
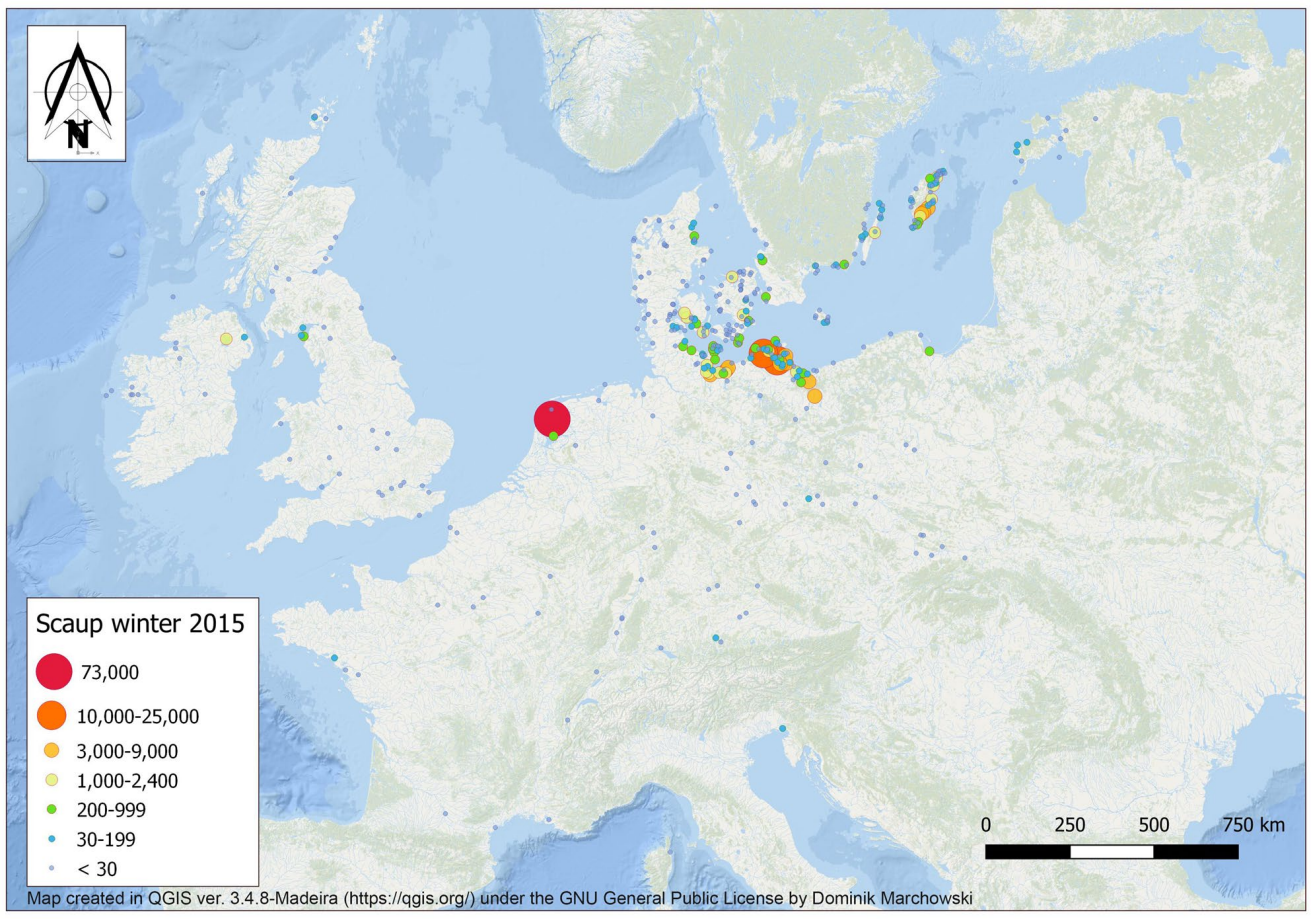
Distribution and abundance of Greater Scaup *Aythya marila*, based on site counts throughout the wintering area in Western and Northern Europe, January 2015. Map created in QGIS ver. 3.4.8-Madeira (https://qgis.org/) under the GNU General Public License by Dominik Marchowski.

**Figure 2:**
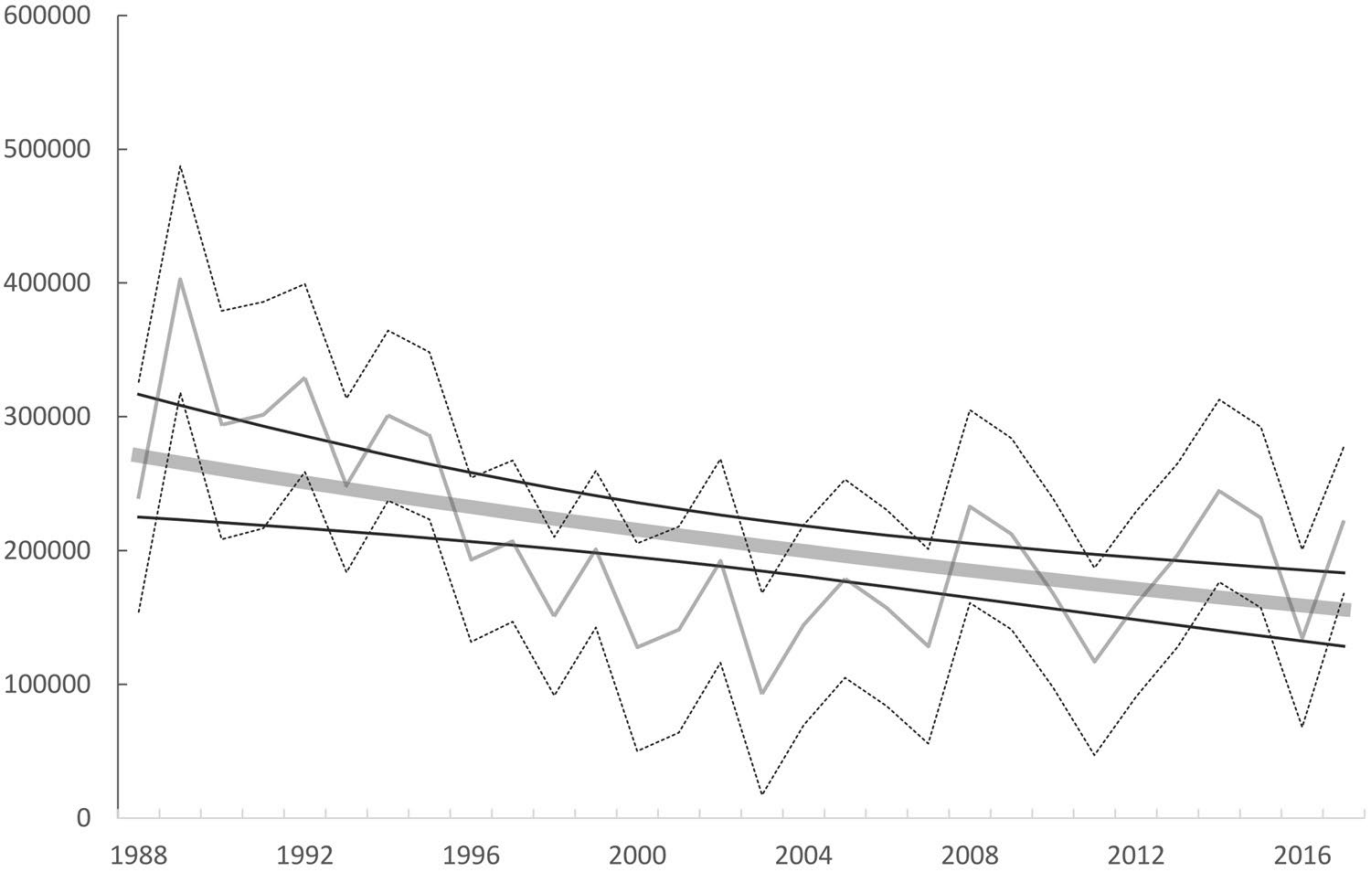
Population trend for Greater Scaup *Aythya marila* wintering in North-West Europe in years 1988–2018, showing a moderate decline, λ = 0.9811 ± 0.0098 95% CI (*P* < 0.001). Grey line—imputed values, black dotted line 95% confidence intervals, grey thick line—modelled trend, black thin lines—95% confidence intervals.

A total of 217,582 Scaup were counted in 22 countries in North-West Europe in January 2015 (Fig. 1); adding modelled numbers from sites with missing counts adjusted the total to 224,715. The modelled trend for 1988–2018 gave a statistically significant annual rate of change of λ = 0.9811 (± 0.0098 95% CI, *P* < 0.01, Fig. 2). The average number modelled in the first four years of the time series (1988–1991) was 309,473 (range 240,103–402,661), compared to 192,498 (134,387–224,715, Fig. 2) in the last four years of monitoring, 38.1% decline in numbers over the time series (or 1.23% yearly, see Table 1 for numbers in each country).

**Table 1:** Estimated mid-winter numbers of Greater Scaup *Aythya marila* from wintering populations in northern and western Europe.

Country	1988–1991	% of population	2015–2018	% of population
Netherlands	170,000	55.0	75,000	39.0
Germany	70,000	22.7	73,000	38.0
Denmark	30,000	9.7	11,000	5.7
Poland	10,000	3.2	12,000	6.2
France	11,000	3.6	200	0.1
United Kingdom	8000	2.6	1800	0.9
Ireland	4000	1.3	1700	0.9
Sweden	4000	1.3	16,000	8.3
Italy	400	0.1	100	0.1
Estonia	50	0.0	1000	0.5
Rest	1550	0.5	500	0.3
N-W Europe total	309,000	100.0	192,300	100.0

Numbers (rounded to the nearest 1000 birds) assigned to individual countries in two periods 1988–1991 and 2015–2018 are given, with each country giving the percentage of the population in relation to the entire biogeographic population.

**Figure 3:**
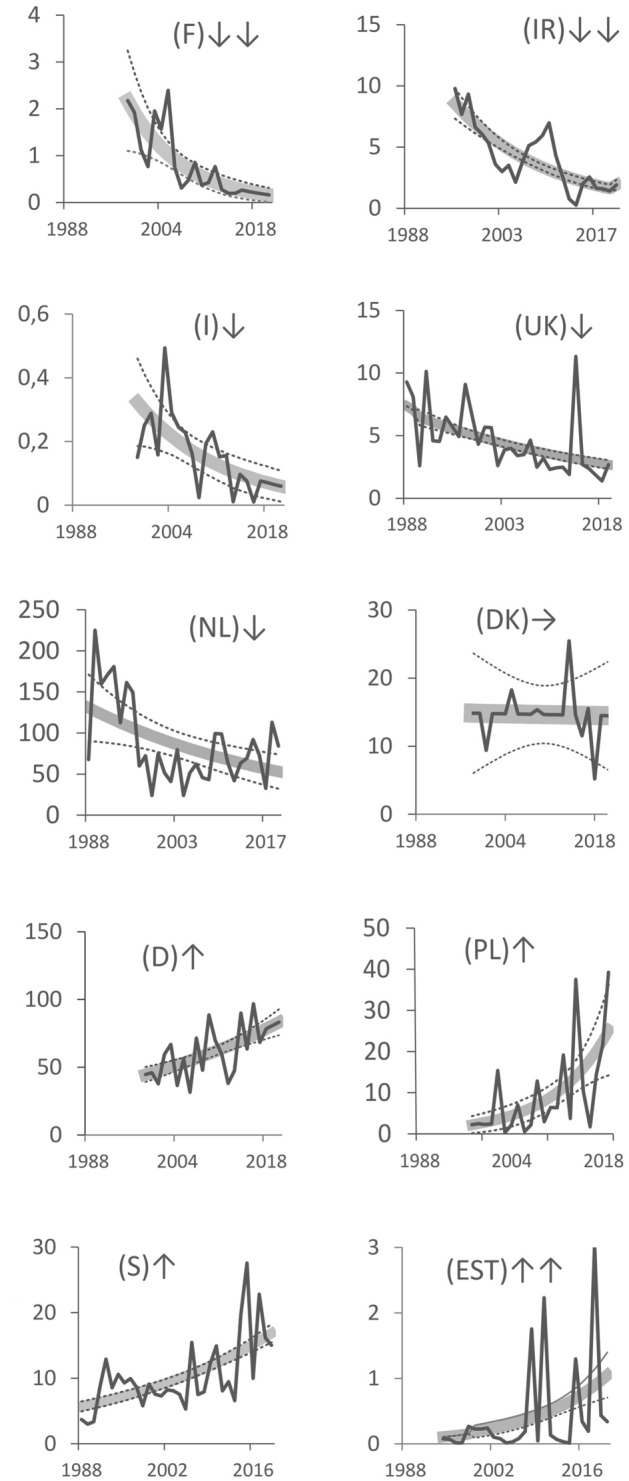
Trends in abundance of Greater Scaup *Aythya marila* in ten countries within the wintering range fitted for data from the year 1988–2019. France (F), Ireland—whole island (IR), Italy (I), The United Kingdom—without Northern Ireland (UK), The Netherlands (NL), Denmark (DK), Germany (D), Poland (PL), Sweden (S) and Estonia (EST). Gray thick line—modelled trend, dotted lines—95% confidence intervals, black line—imputed values. ↓↓—steep decline, ↓—moderate decline, → —stable, ↑—moderate increase, ↑↑—strong increase.

**Figure 4:**
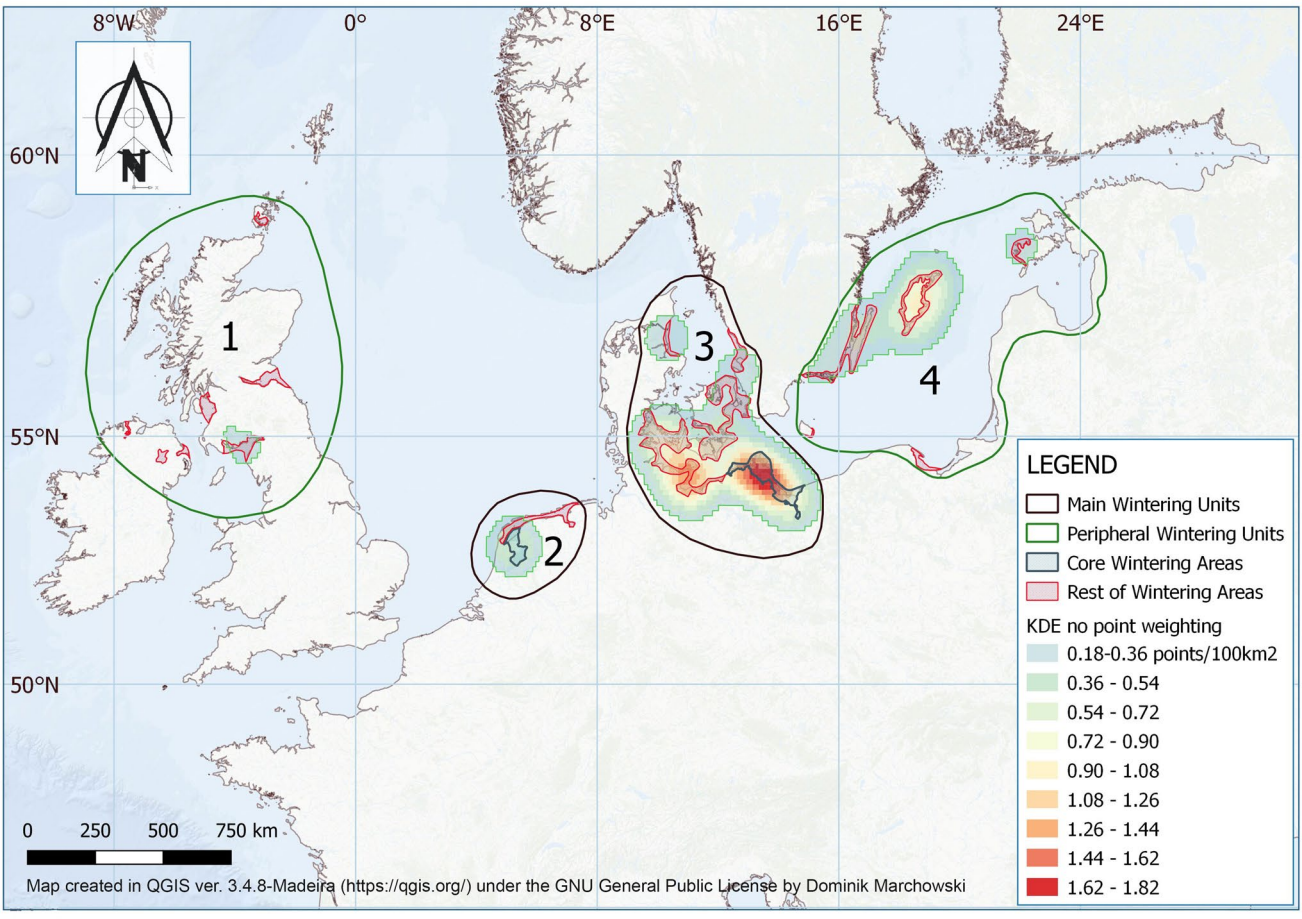
Distribution of the four identified wintering areas for the Greater Scaup *Aythya marila* in Northern and Western Europe based on results of spatial analysis using Kernel Density Estimation to subdivide units within the overall distribution of wintering areas. “Peripheral areas” comprise the wintering population in UK/Ireland—Unit #1, which probably now originates almost entirely from birds breeding in Iceland and Unit #4, which has until now constituted important staging areas but can be expected to become more important wintering areas in the future. Map created in QGIS ver. 3.4.8-Madeira (https://qgis.org/) under the GNU General Public License by Dominik Marchowski.

At the same time, trends in abundance differed between parts of the wintering range (Fig. 3, Table S2). Taken overall, these trends suggest a general shift in the wintering range to the north and east over the duration of the time series. Based on point cluster (Fig. S1) and point displacement (Fig. S2) analysis of Scaup distributions, we identified four main wintering units in North-West Europe, each showing different patterns. Unit#1 (the UK and Ireland) supporting 2% of the entire flyway population. Unit#2 (the Netherlands) supports 39% of the population. Unit#3 (Denmark, Germany, Poland) supports 50%. Unit#4 (Sweden, Estonia and eastern Poland) holds 9% of the population (Fig. 4).

**Figure 5:**
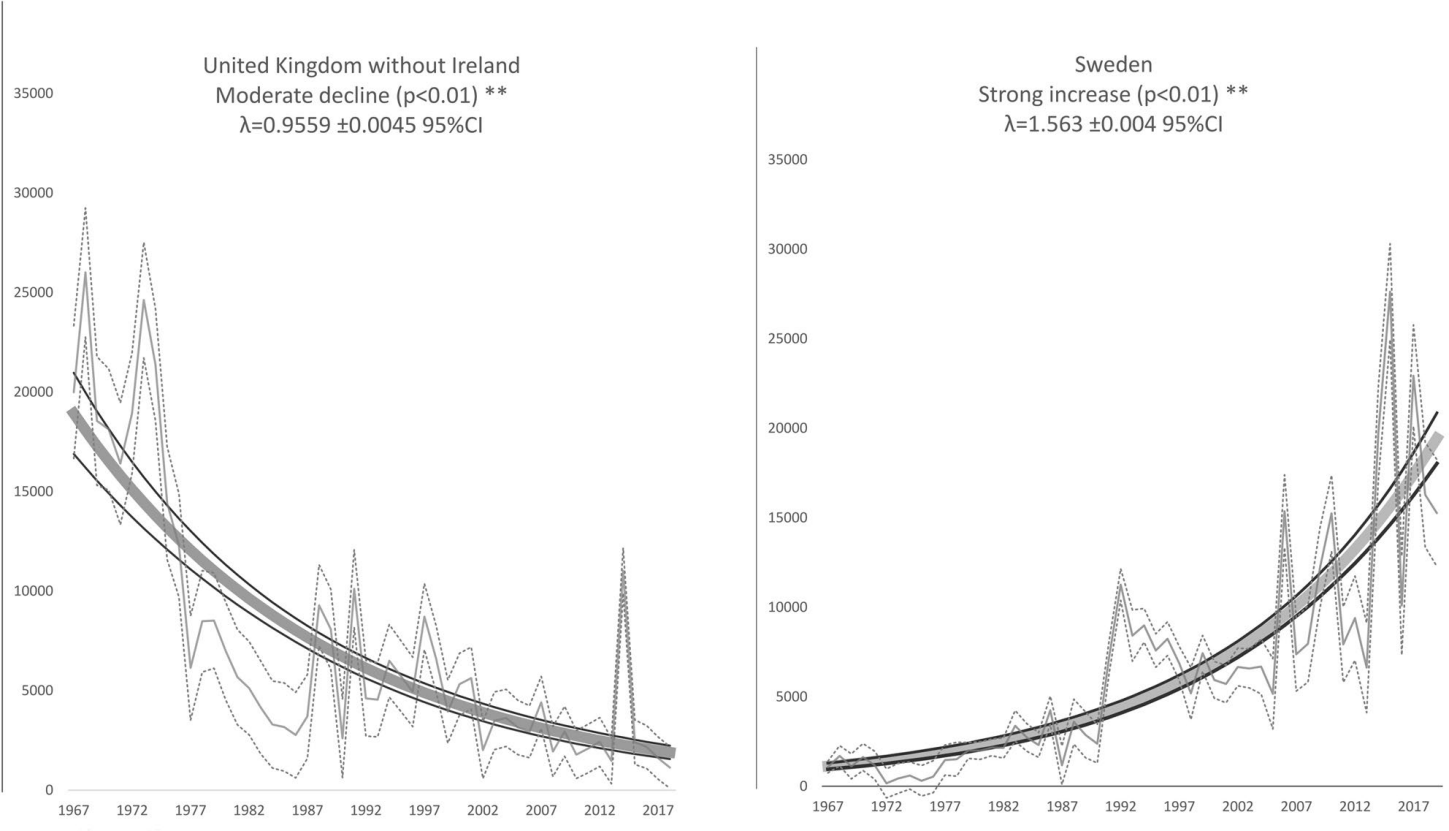
Trends in abundance of Greater Scaup *Aythya marila* at two edge locations of the wintering range: UK and Sweden in the years 1967–2019. Grey thick line—modelled trend, black thin lines—95% confidence intervals, grey thin line—imputed values, black dotted line—95% confidence intervals.

We compared mean numbers from the first four years of monitoring (1967–1970) to the last four years from peripheral areas (the UK 2015–2018 and Sweden 2016–2019). This showed a decrease of 91.2% over this period in the UK, while in Sweden numbers in the first 4 years were 91.5% lower than those from the last four years’ of monitoring (Fig. 5).

### The effect of temperature on the number of birds

The GLM results showed significant effects of both ‘ice’ and ‘year’ on Scaup abundance (*F*_2,85_ = 13.46, *P* < 0.001, explaining 22.3% of the variance in the dependent variable ‘bird no.’). Numbers of Scaup wintering in Sweden, Estonia and Poland generally increased during 1988–2018, but overall, fewer days with temperatures below zero in the first half of January (‘ice’) resulted in more Scaup in these countries. The 'ice' variable (β =  − 0.35, *P* = 0.001) had more influence on Scaup abundance than ‘year’ (β = 0.26, *P* = 0.009).

### Levels of protection in SPAs

Within the wintering range of the studied population, 57 Scaup areas (supporting 30 or more individuals in the years 2015–2019) were identified in eight countries. Analysis of the chi-square tests showed that among all the regular Scaup wintering sites, significantly more of them were protected in the form of Natura 2000 (84.2%). Scaup is the *qualifying species* in just over half of the sites, and there was no statistical difference between the numbers of sites in which the species was/was not a *qualifying species*. Statistically, fewer sites have a management plan—33.3% (Table 2). All areas with significant numbers (i.e. regularly holding 1900 or more wintering individuals), were designated as SPAs with Scaup listed as a *qualifying species*.

**Table 2:** Results of chi-square tests regarding the scope of protection in the form of Natura 2000 sites in which Greater Scaup *Aythya marila* winter.

Variable	n		%		The result of the statistical test—chi-square
	0	1	0	1	
Sites	0	57	0	100	–
SPA/NOT	9	48	15.8	84.2	χ^2^(1) = 26.68;* P* < 0.001
QS/NOT	27	30	47.4	52.6	χ^2^(1) = 0.16; *P* = 0.69
MP/NOT	38	19	66.7	33.3	χ^2^(1) = 6.33; *P* = 0.01

An analysis was carried out to show which areas are protected in the form of Natura 2000 and which are not (SPA/NOT); then checked in which sites Scaup is qualifying species (QS/NOT) and which sites have management plans (MP/NOT).

All of the *core wintering areas* (supporting 67% of the wintering Scaup numbers, Fig. 4) were protected within SPAs. These areas are: IJsselmeer lake in the Netherlands (Unit#2, Core wintering area in Fig. 4) and the lagoons and bays of the southern Baltic coast between Germany and Poland (Unit#3, Core wintering area in Fig. 4). Birds in these areas gather on three SPAs: “IJsselmeer”, “Greifswalder Bodden und südlicher Strelasund” and “Szczecin Lagoon” where Scaup is a *qualifying species* but only “IJsselmeer” has a MP.

## Discussion

International Waterbird Census (IWC) data suggest 309,000 Scaup were wintering in North-West Europe in 1988–1991, compared with 192,300 in 2015–2018, indicating that the number of Scaup in this flyway has declined by 38.1% over 31 years (equivalent to a 30.3% decline over three generations, given a Scaup generation length of 8.2 years). Such a rate of decrease qualifies this population as vulnerable (VU) according to criterion A2(c) of the International Union for Conservation of Nature^24^. Thus, our results confirm the recent attribution of Scaup as a VU on the European Red List^18^. We suggest that the 1% threshold for the North-West Europe population of the Scaup should be revised to 1900.

In addition to the overall decline in abundance, we also show that changes in winter temperature on the eastern and northern edges of the wintering range potentially explain the observed dramatic shift in winter distribution closer to the breeding grounds. Climate change appears to have opened up more wintering sites to Scaup, especially in the more northern and eastern areas where reductions in winter ice cover have made previous staging sites increasingly accessible in winter. This might be expected to have a positive effect on the population, given that Scaup have more potential wintering sites to choose between and that they face a diminished risk from mass starvation because of the reduced probability of unexpected ice cover of potential feeding areas^25^. However, the ultimate causes of shifts in wintering distribution remain unknown and could equally relate to deterioration of food quality in southern and western wintering grounds. At Lake IJsselmeer, the annual changes in the large numbers of wintering Scaup there in the 1980s and 1990s were explained by fluctuations in the abundance of their main prey, Zebra Mussel *Dreissena polymorpha*^26^. The decline in Zebra Mussels in the IJsselmeer lake and its replacement by Quagga Mussels *Dreissena rostriformis bugensis*^27^ resulted in a deterioration in the quality of food resources at the site. These are likely contributory reasons to explain the shift in the centre of gravity of the Scaup wintering grounds to Poland and eastern Germany, although we lack data to determine the magnitude of this effect (Fig. 4, Unit#3). This area now constitutes the most important wintering area for this population, although the detection of Quagga Mussels in this region in 2014^28^ represents a potential threat to the quality of this important wintering ground.

Assuming that some of the birds remain to winter along the migration route on sites formerly only used as stopovers, we can retrospectively infer the migration route of the Scaup population breeding in northern Russia and Fennoscandia (Fig. 1). It would appear that after birds reach the Baltic, they stop in Estonia before traversing the Baltic south-west to Gotland, migrating along the southern coast of Sweden and onwards to the main wintering area in Danish, German and Polish Baltic waters (Unit#3). Some Scaup continue west to reach Unit#2 in the Netherlands, and small numbers continue to reach France and the UK. The small population breeding in Iceland likely winter exclusively in the UK and Ireland, where fewer of the Russian/Fennoscandia population reach in recent winters. The Iceland breeding birds likely constitute a separate biogeographic population, with little contact with the main one discussed here (Fig. 1). Assuming the continuing effects of global warming, we can predict further separation of the two sub-populations and that Unit#4 (Fig. 4), the coast of Gotland and the islands and bays in Estonia will most likely play an increasingly important future role as winter quarters for this species. This is likely to be the case at other sites within eastern Baltic where this species can find suitable habitats.

Our historical analysis has shown that after a period of most rapid decline during 1988–2003, this population could be interpreted as remaining stable during 2003–2018 (Fig. 2). We suspect that this may be partly the result of the significant decrease in the Scaup bycatch in the Netherlands^29–31^. The added mortality from fisheries bycatch represents one of the most important threats to the relatively long-lived Scaup^32^. Evidence showed that drowning mortality was extremely high between 1985 and 1994, when an estimated average of 17,672 birds died annually in fishing gear (6% of the total population of the time), but this has declined since the 2000s^32^. Of all Scaup from this flyway population that drowned in fishing nets in years 1978–1990, up to 65% died at the most important wintering site at the time—the Dutch IJsselmeer^32^. However, our highly uneven knowledge of the extent of the Scaup bycatch throughout its winter range should be taken into account here. Exceptionally detailed estimates from IJsselmeer during the earlier period^14^ contrasts our lack of data or poor estimates from elsewhere, which may result in a bias that implies a greater importance for Scaup bycatch at the IJsselmeer for the population than was actually the case. Current estimates of bycatch levels throughout the flyway suggest that Scaup death in fishing nets has decreased, amounting to *c.*4000 individuals yearly, partly explained by the substantial decrease in the Dutch bycatch^32^.

The second highly important threat to Scaup, perhaps as important as the bycatch, is the deterioration of their food resources. Detailed energy budget studies on Lake IJsselmeer^14^ suggested that foraging Scaup there were operating on the margins of energetic profitability and the limited number of important wintering sites elsewhere suggest that alternative sites are really scarce, implying that food availability at core wintering sites could potentially affect winter survival.

The specialist habitat selection of the Scaup restricts it to a narrow range of habitats during the wintering period where it aggregates in large concentrations, a factor which causes the entire wintering population to concentrate in relatively few locations. Potentially, this makes them more vulnerable at the population level than most other, more dispersed diving duck species. During the January 2015 count, 91% of counted birds were present at 31 locations in five countries (Denmark, Germany, the Netherlands, Poland and Sweden). The four most important locations supported over two-thirds of the total wintering numbers: namely IJsselmeer in the Netherlands, Barther Bodden and Greifswalder Bodden in Germany and Odra river estuary in Poland (Fig. 4). Taken together, these areas have consistently been the most important wintering areas for Scaup over the last 30 years^3,14,20^, with two thirds of the flyway population during winter concentrated within 5300 km^2^ (2000 km^2^ in the Netherlands and 3300 km^2^ in Poland/Germany).

Wintering areas in Germany and Poland also act as stopover sites, so much larger numbers are counted there in autumn and spring migration, with up to 100,000 individuals on the Szczecin Lagoon (*c.*470 km^2^^9^). Similarly, in Estonia, where a few hundred birds winter (Fig. 1), numbers may exceed 100,000 individuals in spring^33^. Therefore, cohesive planning for the effective conservation of the species, requires adequate protection at both the most important wintering sites (analysed in this article) and stopover sites along the entire migration route. During spring migration, extremely large Scaup concentrations can occur in these important sites, which provide for other biological functions such a communal courtship, displaying, pair-bonding etc.^32^. Given that Scaup are among the most vulnerable of diving ducks to bycatch^34^ (constituting more than 50% of diving birds drowned in fishing nets in the Polish Odra Estuary^35^) potentially high mortality during the prelude to the breeding season is likely to have severe adverse effects on the entire population. It is important to remember that this site can simultaneously support up to 75% of the total population^9^ and intensive fishing takes place here with gillnets^35^—the method of fishing recognised as the most dangerous for drowning diving birds in the Baltic Sea^6^.

Other environmental pressures on Scaup are no less serious, but currently less well quantified. Many important wintering areas are situated in estuaries of large rivers that invariably host major sea ports, where large vessels cause disturbance and pollution. Maintenance of shipping channels requires dredging (as in the case of the channel leading to the port of Amsterdam on IJsselmeer in the Netherlands and that serving the port of Szczecin on the Szczecin Lagoon in Poland). Dredging of shallow marine and brackish substrates can disrupt sediment horizons, mobilising suspended material, creating turbidity and disrupting the food resource and the ability of Scaup to forage for their prey. The proximity to human settlements also makes these shallow marine waters attractive to the increasing practice of water sports, kite- and wind-surfing, boating and recreational fishing from boats, which although not a source of direct mortality, contributes to disturbance and displacement of Scaup from favoured areas^36,37^.

### SPAs and effectiveness of protection

The long-term conservation aim for a decreasing *qualifying species*, in accordance with European Union (EU) law (Birds Directive—Council Directive 2009/147/EC), should be to recover them to former level of abundance. To achieve this aim, SPAs should be designated in sites where 1% or more of the biogeographic population regularly occurs. In the case of Scaup, all of such areas are protected in the form of SPA (Table 2). Subsequently, such a SPAs should have a Management Plan (MP) defining the conservation objectives within each site, updated every 6 years. Of the three most important Scaup SPAs in Europe, only the IJsselmeer (NL9803028, Unit#2, Core wintering area, Fig. 4) has a MP for 2013–2017^38^, which described the long term decline (since 1994) in wintering numbers of Scaup in the IJsselmeer and identified the greatest threats for Scaup as declining food resources and disturbance by developing water sports. Although bycatch was conspicuously not listed as a threat, the MP documents previous measures, taken to reduce fishing effort, had resulted from the implementation of another EU Directive—the Water Framework Directive (WFD, Directive 2000/60/EC). The WFD committed EU Member States to achieve good qualitative and quantitative status of all water bodies by 2015^38^. Conservation measures carried out on Lake IJsselmeer over the last 75 years aimed to maintain sustainable fishing did not bring about the intended results on fish stock^39^. However, they may have had a positive effect on reducing bycatch of Scaup from 11,500 killed annually during 1978–1990^32^ to insignificant numbers in the years 2011–2012^31^, which may have contributed to the slowing in the rate of population decline at this time. In the most important wintering area for this flyway populations—the lagoons and bays either side of the German-Polish border, out of ten SPAs forming one coherent area (Fig. 4) only two have MPs. Moreover, the key SPAs within this area that regularly hold the highest Scaup numbers do not have MPs, they are: Greifswalder Bodden und südlicher Strelasund (DE1747402) in Germany and Szczecin Lagoon (PLB320009) in Poland. The Greifswalder Bodden, Teile des Strelasundes und Nordspitze Usedom (DE1747301) Special Area of Conservation (SAC), which overlaps with the Greifswalder Bodden und südlicher Strelasund SPA, was created under the Habitats Directive (Council Directive 92/43/EEC) and has a MP that identifies the threats to Scaup (e.g. from bycatch). However, because MPs for SACs (as against SPAs) are not primarily directed towards bird conservation, there are no specific regulations to limit the current stressors upon Scaup at this site^40^. The existing MPs for two other SPAs (“Vorpommersche Boddenlandschaft und nördlicher Strelasund” and “Dolina Dolnej Odry”) either do not identify main threats to Scaup or fail to impose sufficient conservation measures^41,42^.

Other SPAs that are less important for Scaup within Unit#3 west of the *core wintering area* include Östliche Kieler Bucht (DE1530491) and Ostsee östlich Wagrien (DE1633491), which have MPs identifying the threat from bycatch. This includes a voluntary agreement between the Schleswig Holstein Ministry of the Environment and local fishery associations, under which areas are closed to fishing if “concentrations of ducks” (> 100) are present in the areas. Fishermen have two days to remove their gear after closure. There are rigid legal provisions at these two sites that prohibit fishing with gillnets within 200 m of the shore^43,44^. To date, there is no evidence of a positive effect and reduction of bycatch of diving birds, so we recommend a study of the effectiveness of these provisions.

The shift in the centre of gravity of the wintering population to Germany and Poland highlights the ineffectiveness of conservation measures directed towards Scaup (and other diving birds) there. Despite the existence of SPAs in which the Scaup is specifically protected and evidence of the cost of gillnet bycatch to local diving ducks, the most serious pressure remains unchecked. In 2011–2012, results from research work in the Szczecin Lagoon^37^ recommended the MP proposed reducing the Scaup (and other diving birds) bycatch by spatiotemporal regulation of gillnet fisheries to avoid key areas used by the birds. Unfortunately, the effective solutions to deliver results for bird conservation were considered too far-reaching by fishing interests. The fishing lobby blocked official approval of the MP by government and so these measures were never implemented. Given the high rates of Scaup bycatch, the designation of the area as a SPA offers no effective protection to the species at this site^32^. The effectiveness of SPA designation for a particular species remains ineffective, as long as effective management is not implemented. Given the increasing relative importance of the German/Polish resorts to the species in recent years, the lack of effective measures within these SPAs is becoming more critical to safeguard the conservation of the North-West Europe population of Scaup. Suitably prepared MPs, containing a bycatch monitoring order, would solve this problem, setting bycatch thresholds, according to the recommendations of BirdLife International^45^—1% of natural mortality calculated on the basis of local species abundance. If this value is exceeded, spatiotemporal restrictions on gillnet fishery should be introduced.

Looking to the future, areas that were formerly stopovers are already becoming wintering sites in Sweden and Estonia. Although currently not numerically significant in winter, these sites already hold significant numbers during migration. In the future, satellite areas (Unit#4) have the potential to develop into important wintering grounds and therefore require adequate protection from factors known to affect Scaup survival.

Previous studies show that bycatch in fishing nets is one of the most serious anthropogenic pressures during the non-breeding period for many diving birds^6^, although we cannot exclude the influence of other factors such as food availability and quality^26^ and disturbance from hunting^46^ and water sports^37^. Because the majority of North-West Europe's Scaup winter in relatively few places, conservation interventions at these key sites are particularly important. The shift in wintering distribution poses new challenges for countries increasingly responsible for the conservation of this species in winter. Lack of adequate protection in this region means that these areas may act as *sink habitats* (in the sense of the *source-sink model *^47^). The shift of wintering areas to *sink habitats* exposes an increasing part of the population to the pressures present there. This is not only the case for Scaup but also for a range of other diving bird species. These birds concentrate in the most attractive areas rich in food, often biologically productive transitional waters, where marine and freshwater birds meet in high densities. For this reason, effective conservation measures directed at Scaup will positively impact upon a whole range of other species with similar ecology. This suggests that protection measures taken for the Scaup could also benefit associated marine species in the same areas such as Long-tailed Duck, Velvet Scoter, Common Scoter *Melanitta nigra,* as well as for coastal zone species such as: Tufted Duck, Smew *Mergellus albellus*, and Goosander *Mergus merganser*.

## Supplementary information

Supplementary Information 1.

Supplementary Information 2.

Supplementary Information 3.

## References

[1] Alerstam T, Högstedt G (1982). Bird migration and reproduction in relation to habitats for survival and breeding. Ornis Scand..

[2] Kear J (2005). Duck, Geese and Swans.

[3] Skov H (2011). Waterbird Populations and Pressures in the Baltic Sea.

[4] Durinck J, Skov H, Jensen EP, Pihl S (1994). Important Marine Areas for Wintering Birds in the Baltic Sea.

[5] Mendel B (2008). Profiles of Seabirds and Waterbirds of the German North and Baltic Seas. Distribution, Ecology and Sensitivities to Human Activities Within the Marine Environment.

[6] Žydelis R (2009). Bycatch in gillnet fisheries—an overlooked threat to waterbird populations. Biol. Conserv..

[7] Fox AD, Petersen IK (2019). Offshore wind farms and their effects on birds. Dansk Orn. Foren. Tidsskr..

[8] Amat JA, Green AJ, Hurford C (2010). Waterbirds as bioindicators of environmental conditions. Conservation Monitoring in Freshwater Habitats: A Practical Guide and Case Studies.

[9] Marchowski D, Leitner M (2019). Conservation implications of extraordinary Greater Scaup (*Aythya marila*) concentrations in the Odra Estuary, Poland. Condor.

[10] EBBC (2019). Greater Scaup *Aythya marila*. The European Bird Census Council Atlas of European Breeding Birds.

[11] Umhverfisráðuneytið (1992). Iceland: National Report to UNCED.

[12] Robinson RA, Leech DI, Clark JA (2019). The Online Demography Report: Bird Ringing and Nest Recording in Britain & Ireland in 2018: Scaup *Aythya marila*.

[13] Laursen K (1992). New figures of seaduck winter populations in the Western Palearctic. IWRB Seaduck Bull..

[14] de Leeuw JJ (1999). Food intake rates and habitat segregation of tufted duck *Aythya fuligula* and scaup *Aythya marila* exploring zebra mussels *Dreissena polymorpha*. Ardea.

[15] Lehikoinen A (2013). Rapid climate driven shifts in wintering distributions of three common waterbird species. Glob. Change Biol..

[16] Pavon-Jordan D (2015). Climate-driven changes in winter abundance of a migratory waterbird in relation to EU protected areas. Divers. Distrib..

[17] Wetlands International (2010). Guidance on Waterbird Monitoring Methodology: Field Protocol for Waterbird Counting.

[18] BirdLife International. *Aythya marila*. The IUCN Red List of Threatened Species 2018: e.T22680398A132525108. 10.2305/IUCN.UK.2018-2.RLTS.T22680398A132525108.en (2018).

[19] Pannekoek J, van Strien A (2005). TRIM 3 Manual. Trends and Indices for Monitoring Data.

[20] Marchowski D, Jankowiak Ł, Wysocki D, Ławicki Ł, Girjatowicz J (2017). Ducks change wintering pattern due to changing climate in the important wintering waters of the Odra River Estuary. PeerJ.

[21] Levine N (2015). CrimeStat: A Spatial Statistics Program for the Analysis of Crime Incident Locations (v 4.02).

[22] BirdLife International (2006). Monitoring Important Bird Areas: A Global Framework. Version 1.2.

[23] Wilk T, Jujka M, Krogulec J, Chylarecki P (2010). Important Bird Areas of International Importance in Poland.

[24] IUCN. Standards and Petitions Subcommittee. 2014. In *Guidelines for Using the IUCN Red List Categories and Criteria. Version 11* (Prepared by the Standards and Petitions Subcommittee, 2014). https://cmsdata.iucn.org/downloads/redlistguidelines.pdf.

[25] Suter W, Van Eerden MR (1992). Simultaneous mass starvation of wintering diving ducks in switzerland and the netherlands: a wrong decision in the right strategy?. Ardea.

[26] van Eerden MR, de Leeuw J, Van der Velde G, Rajagopal S, de Vaate AB (2010). How *Dreissena* sets the winter scene for water birds: dynamic interactions between diving ducks and zebra mussels. The Zebra Mussel in Europe.

[27] de Vaate AB, van der Velde G, Leuven RSEW, Heiler KCM, Nalepa TF, Schloesser DW (2013). Spread of the Quagga Mussel (*Dreissena rostriformis bugensis*) in Western Europe. Quagga and Zebra Mussels Biology, Impacts, and Control.

[28] Woźniczka A, Wawrzyniak-Wydrowska B, Radziejewska T, Skrzypacz A (2016). The quagga mussel (*Dreissena rostriformis bugensis* Andrusov, 1897)—another Ponto-Caspian dreissenid bivalve in the southern Baltic catchment: the first record from the Szczecin Lagoon. Oceanologia.

[29] van Eerden MR, Dubbeldam W, Muller J (1999). Sterfte van Watervogels door Visserij met Staande Netten in het IJsselmeer en Markermeer. RIZA rapport 99.060.

[30] Klinge M, Grimm MP (2003). Voor vogels en vissen. Bepaling van de omvang van de vogelsterfte in de staande nettenvisserij in 2002–2003, uitvoering van experimenten met alternatieve visserijtechnieken en evaluatie van maatregelen voor het seizoen 2003–2004.

[31] van den Boogaard B, Krijgsveld KL, van Rijn SHM, Boudewijn TJ (2013). Bijvangst van vogels in staand want in het IJsselmeer en het Markermeer Winter 2012/2013.

[32] Marchowski D, Jankowiak Ł, Ławicki Ł, Wysocki D, Chylarecki P (2020). Fishery bycatch is among the most important threats to the European population of Greater Scaup *Aythya marila*. Bird Conserv. Int..

[33] Kuresoo A, Luigujõe L, Leito A (2008). Technical Assistance for the Deepening and Reconstruction of Shipping Lanes in the West, Environmental Impact Assessment in the Estonian Archipelago Possible Environmental Impact of Planned Activities and Description of the Status of Birds.

[34] Sonntag N, Schwemmer H, Fock HO, Bellebaum J, Gathe S (2012). Seabirds, set-nets, and conservation management: assessment of conflict potential and vulnerability of birds to bycatch in gillnets. ICES J. Mar. Sci..

[35] Psuty I (2017). Developing the Basis for Rational Monitoring of By-catch of Birds for Sustainable Management of Coastal Fishing in the Marine Areas of NATURA 2000.

[36] Robinson JA, Pollitt MS (2002). Sources and extent of human disturbance to waterbirds in the UK: an analysis of Wetland Bird Survey data, 1995/96 to 1998/99. Bird Study.

[37] Ławicki, Ł., Guentzel, S. & Wysocki, D. *Projects of the Managemant Plans for SPAs Natura 2000: the Szczecin Lagoon PLB320009, the Kamien Lagoon and Dziwna PLB320011 and the Odra Mouth River and Szczecin Lagoon PLH320018. Report for the Maritime Office in Szczecin. Project No. POIS.05.03.00-00-280/10*. (ECO-EXPERT Sp.j., Szczecin, 2012).

[38] Beheerplan N2000 (2017). Natura 2000 Beheerplan IJsselmeergebied 2017–2023.

[39] De Leeuw JJ, Dekker W, Buijse AD (2008). Aiming at a moving target, a slow hand fails! 75 years of fisheries management in Lake IJsselmeer (the Netherlands). J. Sea Res..

[40] STALU-VP (2011). Managementplan für das FFH-Gebiet DE 1747–301 Greifswalder Bodden, Teile des Strelasundes und Nordspitze Usedom.

[41] Freitag S (2014). Managementplan für das FFH-Gebiet DE 1542–302 Recknitz-Ästuar und Halbinsel Zingst (in German).

[42] Official Journal West Pomeranian Voivodeship (2017). Ordinance of the Regional Director for Environmental Protection in Szczecin of 27 April 2017 Amending the Ordinance on Establishing a Plan of Conservation Tasks for the Natura 2000 Area Dolina Dolnej Odry PLB320003.

[43] MELUND (2017). Managementplan für das Europäische Vogelschutzgebiet “DE 1530–491 Östliche Kieler Bucht” Teilgebiet “Wasserflächen der Ostsee”.

[44] MELUND (2017). Managementplan für das Europäische Vogelschutzgebiet “DE 1633–491 Ostsee östlich Wagrien” Teilgebiet “Ostseeflächen”.

[45] BirdLife Inernational 2019. *BirdLife Position on Good Environmental Status Threshold Criteria for Descriptor 1: Seabird Bycatch and Population Abundance* (2019). https://portal.helcom.fi/meetings/Incidental%20bycatch%20WS%201-2019-647/MeetingDocuments/BirdLife%20position%20D1criteria_02092019_FINAL.pdf.

[46] Hirschfeld A, Attard G, Scott L (2019). Bird hunting in Europe: an analysis of bag figures and the potential impact on the conservation of threatened species. Br. Birds.

[47] Dias PC (1996). Sources and sinks in population biology. Trends Ecol. Evol..

