# Supplementary material for: Effectiveness of the European Natura 2000 network to sustain a specialist wintering waterbird population in the face of climate change
Source: Sci Rep. 2020 Nov 20;10:20286. doi: 10.1038/s41598-020-77153-4 (PMC7679383; doi:10.1038/s41598-020-77153-4)
Supplement: Supplementary file 1 — Supplementary Information 1. [file 41598_2020_77153_MOESM1_ESM.docx]

Manuscript title: Effectiveness of the European Natura 2000 network to sustain a specialist wintering waterbird population in the face of climate change

Author list: Dominik Marchowski, Łukasz Ławicki, Anthony D. Fox, Rasmus Due Nielsen, Ib K. Petersen, Menno Hornman, Leif Nilsson, Fredrik Haas, Johannes Wahl, Jan Kieckbusch, Hans W. Nehls, Neil Calbrade, Richard Hearn, Włodzimierz Meissner, Niamh Fitzgerald, Leho Luigujoe, Marco Zenatello, Clemence Gaudard, Sven Koschinski.

Supplementary Information

Table S1. TRIM categories and criterion

| Trend category | Significance | Criterion |
| --- | --- | --- |
| Strong increase | Increase significantly more than 5% per year. | Lower limit of CI >1.05. |
| Moderate increase | Significant increase, but not significantly more than 5% per year. | 1.00 < lower limit of CI <1.05 |
| Stable | No significant increase or decline, and it is certain that trends are less than 5% per year. | CI encloses 1.00 but lower limit >0.95 and upper limit <1.05 |
| Uncertain | No significant increase or decline, but not certain if trends are less than 5% per year. | CI encloses 1.00 but lower limit <0.95 or upper limit >1.05 |
| Moderate decline | Significant decline, but not significantly more than 5% per year. | 0.95 < upper limit of CI <1.00 |
| Steep decline | Decline significantly more than 5% per year | Upper limit of CI <0.95 |

Table S2. Trends in abundance of Greater Scaup *Aythya marila* in the most important countries within the wintering range of the North-West Europe population. All available data has been used. ↓↓ - Steep decline, ↓ - Moderate decline, → - Stable, ↑ - Moderate increase, ↑↑ - Strong increase.

| Country | Period of time | Trend | Pop. growth rate (λ) | 95% CI |
| --- | --- | --- | --- | --- |
| France | 2000-2018 | ↓↓ | 0.8835 | 0.0553 |
| Ireland | 1995-2018 | ↓↓ | 0.9303 | 0.018 |
| Italy | 1992-2015 | ↓ | 0.9507 | 0.0157 |
| United Kingdom | 1967-2018 | ↓ | 0.9559 | 0.0047 |
| Netherlands | 1988-2018 | ↓ | 0.9704 | 0.0194 |
| Denmark | 2000-2018 | → | 0.9988 | 0.0466 |
| Germany | 2000-2016 | ↑ | 1.0302 | 0.0106 |
| Poland | 2000-2018 | ↑ | 1.0942 | 0.0652 |
| Estonia | 1993-2019 | ↑↑ | 1.0939 | 0.0351 |
| Sweden | 1967-2019 | ↑↑ | 1.0563 | 0.0392 |

Appendix 1. Table S3. List of areas important as wintering grounds for Greater Scaup *Aythya marila* in northern and western Europe.

S3 table legend: column A - Country; column B - name of site where Scaup herds were observed greater than or equal to 30 individuals; C - is the site protected under the Natura 2000 network (yes or not); D - Natura 2000 area code; E - area of the Natura 2000 area in km2; F - area of the Natura 2000 area in hectares; G - Date classifying the area as Special Protection Area Natura 2000; H - Date of the last update Standard Data Form for the Natura 2000 site; I - is Greater Scaup a qualifying species for a given area (yes / no); J - does the area have a management plan (yes / no); K - the maximum number of individuals entered in the Standard Data Form; L - Determination of the area value for Greater Scaup on a scale from A to D, where A - highest value, D - insignificant; M - determination of what kind of population resides in the Natura 2000 area, w - wintering, c - concentration.


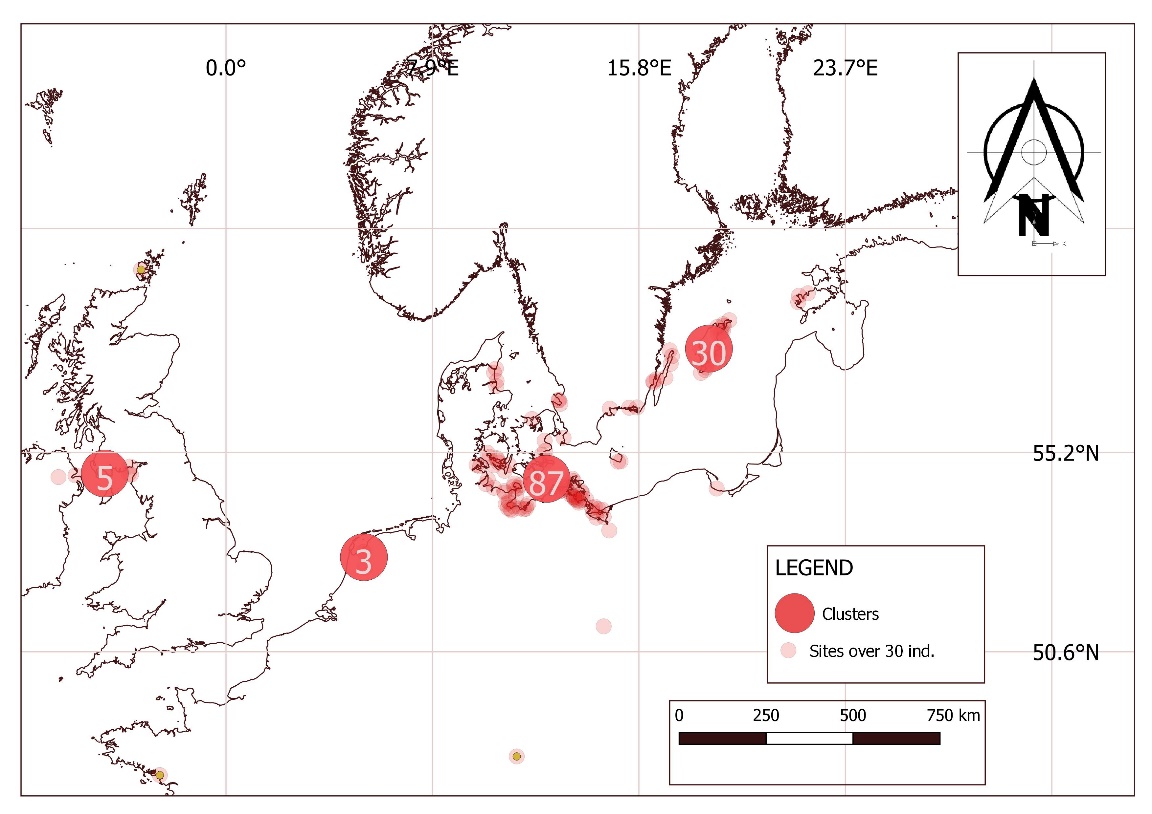


Fig. S1. Distribution of the four most important clusters of Greater Scaup *Aythya marila* wintering grounds, based on coordinated count in 2015. Function used: point cluster in Quantum GIS 3.4.8 software, map scale 1: 12,500,000. Map created in QGIS ver. 3.4.8-Madeira (<https://qgis.org/>) under the GNU General Public License by Dominik Marchowski.


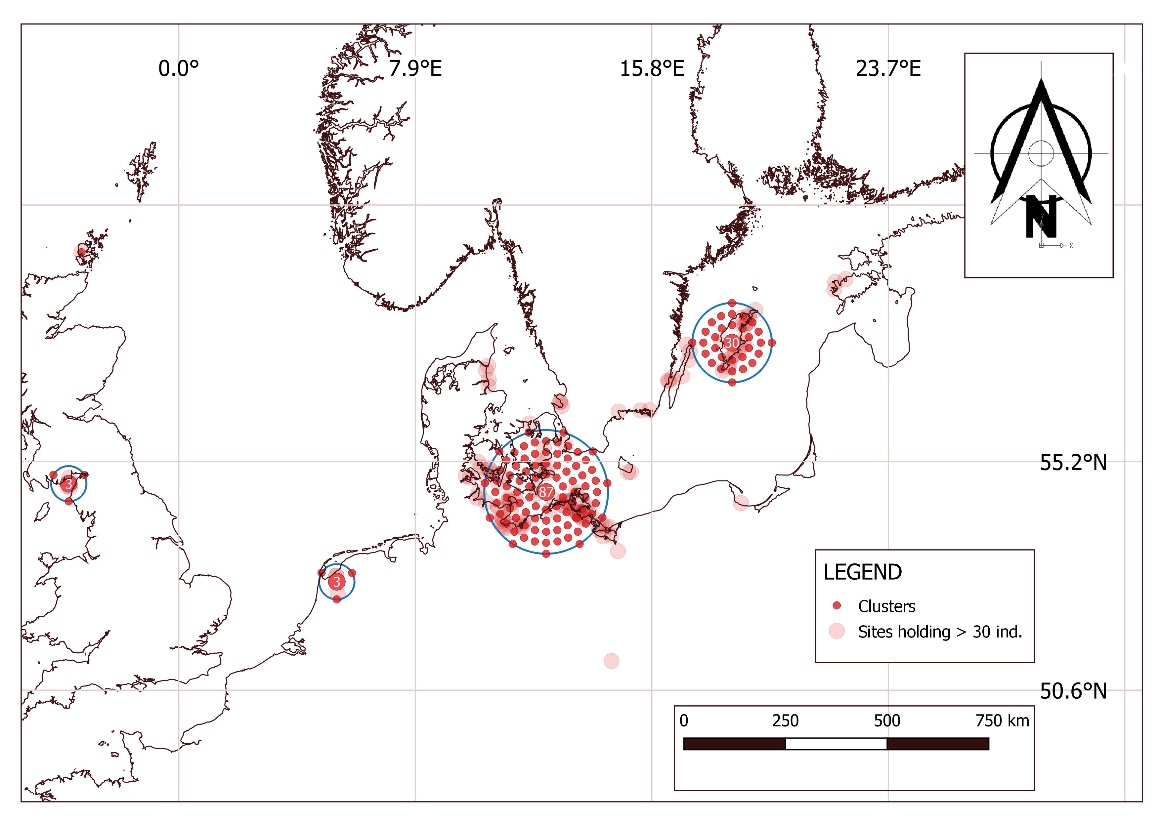


Fig. S2. Distribution of the four most important clusters of Greater Scaup *Aythya marila* wintering grounds, based on coordinated count in 2015. Function used: point displacement in Quantum GIS 3.4.8 software, map scale 1: 12,500,000. Map created in QGIS ver. 3.4.8-Madeira (<https://qgis.org/>) under the GNU General Public License by Dominik Marchowski.

Appendix 2. Packed text files. Raw data used for statistical calculations, prepared in a format for use in TRIM software (Trends and Indices for Monitoring data, ver. 3.54).
